# Functional Characterization of the Human Islet Microvasculature Using Living Pancreas Slices

**DOI:** 10.3389/fendo.2020.602519

**Published:** 2021-01-15

**Authors:** Luciana Mateus Gonçalves, Joana Almaça

**Affiliations:** Division of Endocrinology, Diabetes and Metabolism, Department of Medicine, University of Miami Miller School of Medicine, Miami, FL, United States

**Keywords:** pancreatic islet, microvasculature, pericytes, smooth muscle cells, pancreas slices, vasomotion

## Abstract

Pancreatic islets are clusters of endocrine cells that secrete different hormones to regulate blood glucose levels. Efficient hormone secretion requires a close interaction of endocrine cells with their vascular system. Islets receive blood through feeding arteriole(s) that branch into capillaries made of endothelial cells covered by pericytes. While a lot is known about rodent islet blood vessels, the structure and function of the human islet microvasculature has been less investigated. In this study, we used living pancreas slices from non-diabetic human donors to examine the function of human islet blood vessels. Living human pancreas slices were incubated with a membrane permeant calcium indicator and pericytes/smooth muscle cells were visualized with a fluorescent antibody against the mural cell marker NG2 proteoglycan. By confocal microscopy, we simultaneously recorded changes in the diameter of lectin-labeled blood vessels and cytosolic calcium levels in mural cells in islets. We tested several stimuli with vasoactive properties, such as norepinephrine, endothelin-1 and adenosine and compared human vascular responses with those previously published for mouse islet blood vessels. Norepinephrine and endothelin-1 significantly constricted human islet feeding arterioles, while adenosine dilated them. Islet capillaries were less responsive and only 15–20% of the mouse and human islet capillary network showed vasomotion. Nevertheless, in these responsive regions, norepinephrine and endothelin-1 decreased both mouse and human islet capillary diameter. Changes in islet blood vessel diameter were coupled to changes in cytosolic calcium levels in adjacent mouse and human islet mural cells. Our study shows that mural cells in islets are the targets of different regulatory mechanisms of islet blood perfusion. Several alterations of the human islet microvasculature occur during diabetes progression. Elucidating their functional consequences in future studies will be critical for our understanding of disease pathogenesis.

## Introduction

Pancreatic islets are endocrine mini-organs rich in blood vessels (1, 2). Close interactions between islet endocrine cells and vascular cells are established early on during development and maintained throughout life, by providing mutual trophic and functional support (2–7). Indeed, as any other endocrine organ, islets depend on their blood vessels to function properly (8). Defects in the islet microvasculature can lead to diabetic phenotypes (9). Several studies have reported structural alterations of the human islet microvasculature and composition of the extracellular matrix during type 1 and type 2 diabetes (10–15). However, the functional consequences of these alterations are hard to assess in humans. In animals, altered islet blood flow, for instance, is observed in different rodent models of disturbed glucose homeostasis (16, 17). Microvascular dysfunction and abnormal regulation of islet blood flow could compromise exchanges between endocrine cells and the circulation, resulting in defective hormone secretion as previously suggested (18, 19).

The mouse is an extensively used and valuable animal model in medical research. However, previous studies have shown that in human islets the microvascular niche differs significantly from that of the mouse. Not only human islets have less and shorter capillaries than mouse islets (13, 20), but also their vessels are surrounded by a double basement membrane (21) and a denser layer of connective tissue exists in their perivascular space (22). Another important difference between human and mouse islets is that human beta cells produce an amyloidogenic peptide (islet amyloid polypeptide, IAPP) that accumulates next to capillaries within the human islet parenchyma (23, 24). These anatomical differences in the islet microcirculation between the two species prompted us to examine whether blood vessels in human islets would also be regulated differently than those in mouse islets.

To examine microvascular function in human islets, we adopted the pancreatic slice technique (25). In pancreas slices, islet vascular networks are preserved, and their function can now be studied within the native pancreatic environment (11, 20). We have previously used this platform to monitor microvascular responses in mouse islets *ex vivo* (11, 22). Similar to mouse pancreas slices, living human pancreas slices can also be produced from small pancreas pieces and used in physiological experiments acutely after slicing (26) or after long-term culture [at least 10 days (27)]. We have recently performed dynamic hormone secretion assays and imaged intracellular calcium levels in living human pancreas slices to characterize endocrine cell function in individuals at different stages of type 1 diabetes (26). Living human pancreas slices have also enabled us to study how endocrine cells communicate with other cells in the human islet microenvironment such as islet resident macrophages (28). In this study, we used living pancreas slices from non-diabetic donors to investigate the cellular mechanisms that control vasomotion in human islets. By confocal imaging, we simultaneously recorded changes in islet blood vessel diameter and cytosolic calcium levels in mural cells. To the best of our knowledge, this is the first functional characterization of human islet blood vessels and comparison with mouse islet vascular responses.

## Methods

### Human Organ Donors

We obtained human living pancreas slices from de-identified cadaveric donors (from the head of the pancreas, n = 6 non-diabetic individuals, male and female, ages from 20–59 years old; information on the donors used in this study is provided in **Table S1**) from the Network of Pancreatic Organ Donors with Diabetes (nPOD) or sliced locally at the Diabetes Research Institute (University of Miami). Slices produced by nPOD were shipped overnight from Gainesville to Miami and used 3 h after arrival, while slices produced locally were cultured overnight and used the day after (26, 27).

### Tissue Viability

Pancreatic slices were incubated with calcein-AM (to label live cells in green) and ethidium homodimer-1 (labels dead cells in red). Addition of both reagents was done according to the manufacturer’s recommendations as part of the Live/Dead viability/cytotoxicity kit for mammalian cells [Invitrogen, Carlsbad, CA, Cat# L3224; (27)]. Endocrine cell calcium responses to KCl depolarization (25 mM; protocol of loading with calcium indicator is below) were used as an additional readout of tissue viability. Only viable and responsive tissue was used in subsequent experiments.

### Confocal Imaging of Living Pancreas Slices

Living human pancreas slices were incubated with Fluo4-AM (final concentration 6.3μM, Invitrogen, cat. nr. F14201) and DyLight 649 lectin from *Lycopersicon Esculentum* (final concentration 3.3μg/mL, VectorLabs, cat. nr. DL1178) for 1 h in 3 mM glucose solution prepared in HEPES buffer (125 mmol/l NaCl, 5.9 mmol/l KCl, 2.56 mmol/l CaCl_2_, 1 mmol/l MgCl_2_, 25 mmol/l HEPES, and 0.1% BSA [w/v], pH 7.4) supplemented with aprotinin (25 KIU, MilliporeSigma, cat. nr. A6106) at room temperature and in the dark. To identify pericytes *in situ*, slices were incubated for 2 h with a fluorescent-conjugated antibody against NG2 (final dilution 1:50, R&D Systems, cat. nr. Fab2585R). After incubation, living pancreas slices were placed on a coverslip in an imaging chamber (Warner instruments, Hamden, CT, USA) and imaged under an upright confocal microscope (Leica TCS SP5 upright; Leica Microsystems, Wetzlar, Germany). The chamber was continuously perfused with HEPES-buffered solution containing 3 mM glucose and confocal images were acquired with LAS AF software (Leica Microsystems) using a 40X water immersion objective (NA 0.8). We used a resonance scanner for fast image acquisition to produce time-lapse recordings spanning 50–100 μm of the slice (z-step: 5–10 μm, stack of 10–15 confocal images with a size of 512 × 512 pixels) at 5 seconds resolution (xyzt imaging). Fluo-4 fluorescence was excited at 488 nm and emission detected at 510–550 nm, DyLight 649 labeled tomato lectin was excited at 638 nm and emission detected at 648–690 nm.

From each human donor, a group of slices was incubated with Fluo4 and lectin and another group of slices was incubated with Fluo4 and NG2-alexa647, to account for potential effects of antibody binding and change in cell physiology. Mural cells responded similarly with or without antibody labeling.

We recorded changes in [Ca^2+^]_i_ and blood vessel diameter induced by norepinephrine (20 μM; applied for 3 min), endothelin-1 (10 nM, applied for 5 min) and adenosine (50 μM, applied for 4 min). To quantify changes in [Ca^2+^]_i_, we drew regions of interest around individual islet pericytes and measured the mean Fluo4 fluorescence intensity using ImageJ software (http://imagej.nih.gov/ij/). Changes in fluorescence intensity were expressed as percentage over baseline (ΔF/F). The baseline was defined as the mean of the three first values of the control period of each recording [i.e., in non-stimulatory, basal glucose concentration conditions (3 mM)]. Blood vessels were labeled with DyLight-649 and we could image vessel borders in slices. Quantification of vessel diameter was done as previously described (11). Briefly, we drew a straight-line transversal to the blood vessel borders (**Figure 3A’** and **A”**) and used the “reslice” z-function in ImageJ to generate a single image showing the changes in vessel diameter over time (xt scan; temporal projections shown in **Figures 3C–E**). Noise from reslice images was removed using a median filter (radius = 1 pixel). We drew another line on the xt scan (resliced) image and, using the “plot profile” function, we determined the position of the pixels with the highest fluorescence intensity and considered these the vessel borders. Vessel diameter was calculated by subtracting these two position values. To determine the extent of constriction/dilation, we pooled data on responsive capillaries from different islets from different donors and calculated the relative change in diameter (as fraction of initial vessel diameter). We estimated the proportion of “responsive capillaries” by dividing the area of the lectin-labeled capillaries that responded to a certain stimulus by the total area of the islet capillary network.

### Immunohistochemistry

Small pieces of pancreatic tissue were fixed overnight with 4% PFA and then placed in PBS. Slices were incubated in blocking solution (PBS-Triton X-100 0.3% and Universal Blocker Reagent; Biogenex, San Ramon, CA) for 3 h. Thereafter, slices were incubated for 48 h (20°C) with primary antibodies diluted in blocking solution. We immunostained pericytes (NG2, 1:50–1:100), endothelial cells (CD31, 1:25), smooth muscle cells (αSMA, 1:250), beta cells (insulin, 1:2000). Immunostaining was visualized conjugated secondary antibodies (1:500 in PBS; 16 h at 20°C; Invitrogen, Carlsbad, CA). Cell nuclei were stained with dapi. Slides were mounted with Vectashield mounting medium (Vector Laboratories) and imaged on an inverted laser-scanning confocal microscope (Leica TCS SP5; Leica Microsystems) with LAS AF software using a 63X oil immersion objective (NA 1.4). To quantify colocalization between αSMA and NG2 immunostainings, we calculated Mander’s coefficients in confocal images using the ImageJ plugin “JACoP”: Just Another Co-localization Plugin.

### Statistical Analyses

For statistical comparisons we used Prism 7 (GraphPad software, La Jolla, CA) and performed Student’s t tests (paired and unpaired) or One sample t test. *p* values < 0.05 were considered statistically significant (indicated with an *). Throughout the manuscript we present data as mean ± SEM.

## Results

### Pericytes Cover Human and Mouse Islet Capillaries and Express αSMA

**Figure 1:**
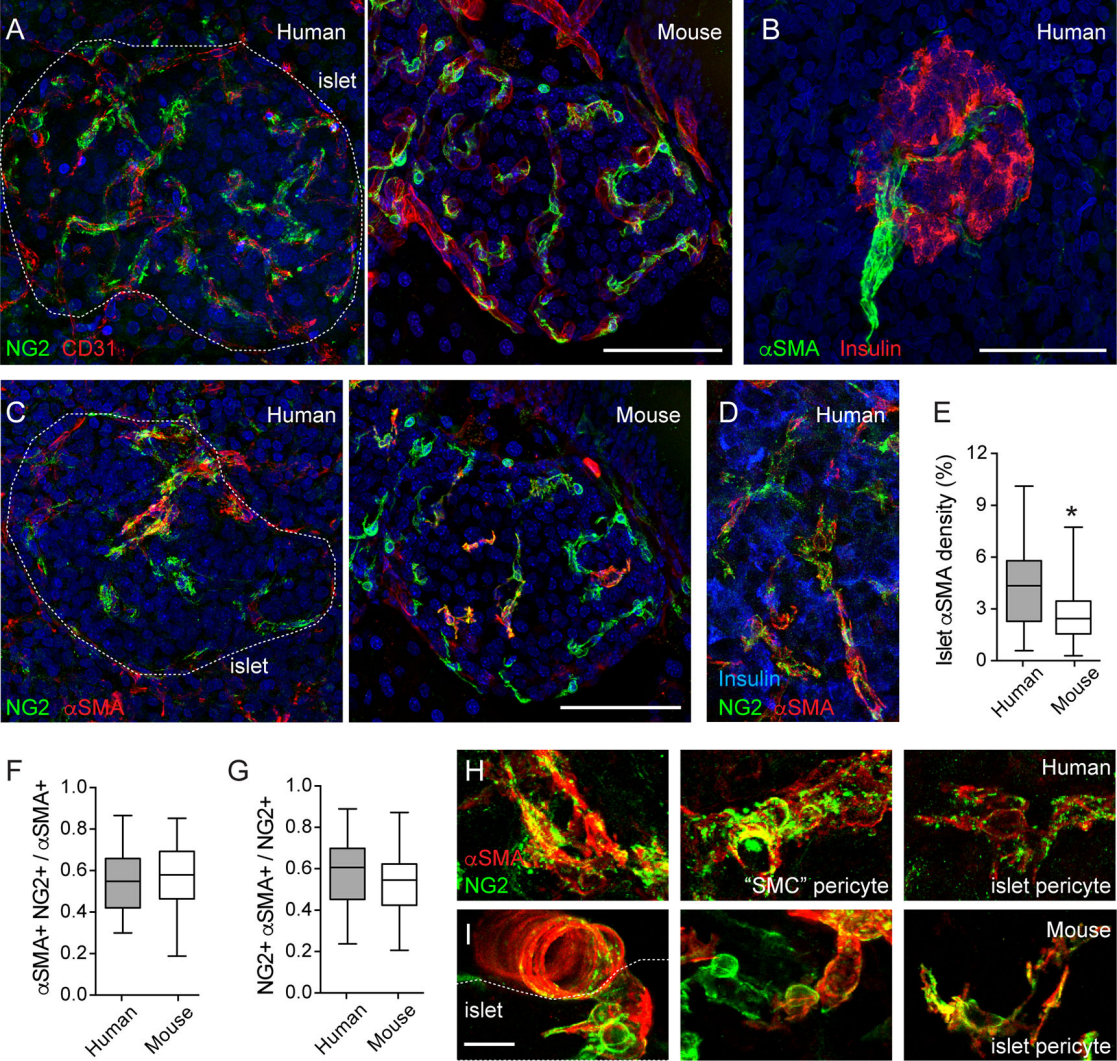
Pericytes cover human and mouse islet capillaries and express αSMA. **(A)** Maximal projection of confocal images of a human (left panel; 16-year-old individual) and a mouse islet (right panel; 2 months old) in pancreatic sections immunostained for pericytes (neuron-glial antigen 2 (NG2), green) and endothelial cells (CD31, red). Pericytes cover mouse and human islet capillaries. Their densities in islets are higher than in surrounding exocrine tissues. **(B)** Maximal projection of confocal images of a human islet immunostained for insulin (red) and smooth muscle actin alpha isoform (αSMA, green). The donor was a 16-year-old individual. Scale bar = 50 μm. **(C)** Maximal projections of confocal images of human (left panel; 16 year old) and a mouse islet (right panel; 2 months old) in pancreatic sections immunostained for pericytes (NG2, green) and αSMA (red). **(D)** Maximal projections of confocal images of a region within a human islet immunostained for NG2 (green), αSMA (red) and insulin (blue). Human donor was a 44-year-old individual. **(E)** Quantification of the islet αSMA density, which is the % of αSMA immunostained area divided the total islet area (N = 24 human islets from 7 non-diabetic human donors (male and female; ages: 15–55 years old); N = 37 mouse islets from 6 C57BL6 mice; 2–18 months old). *p < 0.05 (unpaired t-test). **(F**, **G)** Mander’s (M1 and M2) coefficients reflecting the colocalization between NG2 and αSMA immunostainings. **(F)** is the fraction of αSMA-positive immunostaining that colocalizes with NG2 and **(G)** is the fraction of NG2 -positive immunostaining that colocalizes with αSMA. Panel G has been partially published in (11). We analyzed 21 human islets from 7 non-diabetic human donors (male and female; ages: 15-55 years old) and 31 mouse islets from 6 C57BL6 male mice (2–18 months old). **(H**, **I)** Zoomed images of NG2 (green) and αSMA (red) labeled cells in human **(H)** and mouse islets **(I)** Different types of pericytes can be found in human islets. We named “SMC” pericytes those cells with more circumferential cytoplasmic processes found on the surface of human islet feeding arterioles (left and middle panels, **H**) to distinguish them from pericytes that were present in the islet parenchyma (islet pericytes, right panels in H and I). “SMC” pericytes and some of human and mouse islet pericytes express αSMA. Scale bars = 50 μm **(A**–**C)** and 10 μm **(H**, **I)**.

Pericytes, the mural cells of the microcirculation, are part of the mouse and human islet microenvironment (5, 11, 29, 30). Pericytes are complex cells (31). The unambiguous distinction of pericytes from other perivascular cells requires inspecting their basement membrane at the ultrastructural level (32). However, pericytes can be identified in tissue sections using antibodies against pericyte surface markers, such as neuron-glial antigen 2 (NG2) and platelet-derived growth factor receptor-beta (PDGFRβ). Pericytes have a typical bump-on-a-log morphology: a cell body with a prominent nucleus and cytoplasmic processes on the surface of capillaries (31, 33) (**Figures 1H, I**). Following this classification, we have previously reported that mouse and human islet capillaries are covered with pericytes and estimated an average pericyte: endothelial cell ratio of 1:3–1:2 for mouse and human islets, respectively (11). Pericytes interact closely with islet endothelial cells and are present on straight parts of islet capillaries and at capillary branch points (**Figure 1A**). Interestingly, pericyte density in islets is higher than in the surrounding exocrine tissue [**Figure 1A**; (30)]. Pericytes in mouse and human islets also express PDGFRβ, although expression of this receptor is not limited to pericytes in human islets (10).

**Figure 2:**
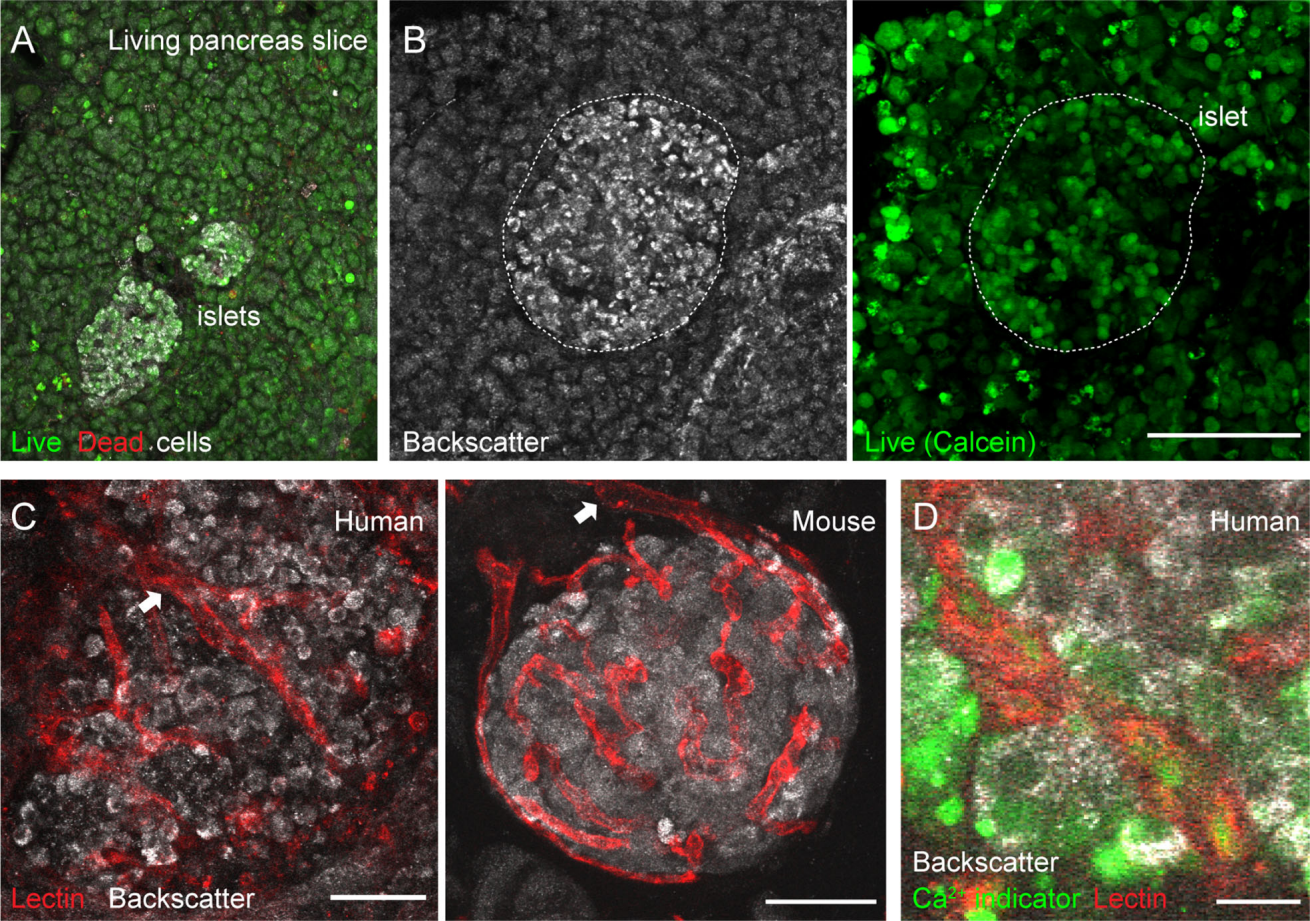
Vascular trees are preserved in living human and mouse pancreas slices. **(A**, **B)** Maximal projection of confocal images of living human pancreas slices stained with calcein-AM (to visualize live cells, green) and with ethidium homodimer-1 (to visulize dead cells, red). Islet endocrine cells can be seen using the backscatter signal (white, left panel in **B**). The majority of the pancreatic tissue is alive in slices. **(C)** Vascular trees in living human (left panel) and mouse (right panel) living pancreas slices. Human slices were incubated with a fluorescent lectin (from *Lycopersicon esculentum*) to label blood vessels (red), while the lectin was injected intravenously in the mouse before extracting the pancreas. The islet microvasculature is preserved in living pancreas slices. Arrows point to islet feeding arterioles. **(D)** Living human pancreas slices can be incubated with membrane permeable calcium indicators (e.g., Fluo4, green) and fluorescent lectins (red) to simultaneously monitor changes in islet endocrine cell or vascular cell activity. Scale bars = 40 μm **(A**–**C)** and 10 μm **(D)**.

Pericytes in mouse islets have contractile properties (11). To determine if pericytes in human islets also help regulating islet capillary diameter, we examined in more detail pericytic expression of α-smooth muscle actin (αSMA). αSMA is expressed by cells of smooth muscle cell lineages, allowing us to visualize arterioles that feed into pancreatic islets (**Figures 1B, C**). In most human islets only one feeding arteriole would be seen, in line with previous findings (34). αSMA positive cells are also present within the human and mouse islet parenchyma (**Figures 1C, D**). The density of αSMA immunostaining inside human islets is higher than what is found in mouse islets (**Figure 1E**), as previously reported (35). Around 50% of the αSMA positive immunostaining colocalizes with NG2 (**Figures 1C, D, F**). This fraction tends to decrease with donor age (data not shown). Importantly, around 60% of the NG2 positive cell population expresses αSMA [**Figures 1C, D, G**; (11)]. In human islets, a subset of these double positive cells are found tightly wrapping the capillary end of the feeding arteriole (**Figures 1C, H**). These pericytes have a different morphology than those found in the islet parenchyma. Indeed, it is known that pericyte morphology depends on their location in the capillary bed: more circumferential cytoplasmic processes at the arteriole end, more longitudinal processes in the middle, and a stellate morphology toward the venule end of the capillary bed (33). Given the circumferential morphology of their cytoplasmic processes and the fact that they are associated with an arteriole instead of a capillary (vessel diameter of 8–10 μm), this type of islet pericytes resembles more a transitional state toward smooth muscle cells. Therefore, in this study, we named them “SMC” pericytes, while pericytes in the human islet parenchyma and covering capillaries are referred to as human islet pericytes (**Figure 1H**). In mouse islets, the morphology of pericytes (and of blood vessels, see **Figure 2C**) is more homogenous and, thus, we refer to them all as mouse islet pericytes (**Figure 1I**). To summarize, a significant number of mouse and human islet pericytes expresses αSMA, as previously reported for pericytes in the central nervous system (11, 36). These data indicate that pericytes in human and mouse islets may participate in blood flow regulation, but in human islets, they are more heterogenous in terms of morphology and contractile protein expression.

### Vascular Trees are Preserved in Living Human and Mouse Pancreas Slices

Isolated islets are the most common research material used to study the physiology of pancreatic islet cells in humans. However, islets, similarly to other endocrine organs (37), lose a significant amount of their vascular cells during the isolation process and with culture (38). To overcome this obstacle and be able to study vascular responses in human islets, we adopted the pancreatic slice technique (25, 39). In pancreas slices, the different cellular components of the islet vascular network are preserved (20), and we have used this platform to perform a detailed functional analysis of mouse islet microvascular responses within the native pancreatic environment (11). Living human pancreas slices produced from fresh tissue samples obtained from the nPOD program have been used by us to compare endocrine cell function in non-diabetics with that of individuals at different stages of type 1 diabetes (26). In the current study, we have used pancreas slices from six organ donors procured either by nPOD or received locally at the Diabetes Research Institute (**Table S1**). Tissue slices were produced within few hours after organ arrival and experiments performed 24 h after slicing. In pancreas slices, the morphology of both exocrine and endocrine tissue compartments is preserved and islet endocrine cells can be distinguished from the surrounding tissue due to the strong light scattering properties of mature secretory granules [**Figure 2B**; (40)]. The vast majority of cells within pancreas slices is viable and, except for the cutting surface, few dead cells are detected (**Figure 2A**). Previous studies had already shown no signs of increased tissue inflammation or immune cell infiltration in islets from non-diabetic organ donors associated with slicing of living tissue (26, 28).

Fluorescent lectins (e.g., from *Lycopersicon esculentum*) that bind to glycoproteins located in the endothelial basement and plasma membranes can be used to visualize blood vessels *ex vivo*. While in mice these lectins can be injected intravenously before sacrificing the animals, living human pancreas slices have to be incubated with them after tissue slicing. In any case, lectin labeling allows us to visualize islet vascular trees (**Figure 2C**). Islet feeding arterioles, which are larger blood vessels that branch off into smaller vessels or capillaries, can be seen penetrating the human islet parenchyma or at the border in mouse islets (**Figure 2C**). Notably, mouse and human islet capillaries do not collapse during slicing, and a lumen is still present in many of them, allowing us to monitor changes in islet vessel diameter by confocal microscopy. Unlike mouse slices, which can be produced from transgenic animals that express genetically encoded fluorescent indicators in specific cell types [e.g., from transgenic mice that express GCaMP3 in pericytes; see **Figure 5** and (11)], human slices have to be incubated with indicators that report on cellular activity. For instance, membrane permeable calcium indicators (such as Fluo4) allow us to monitor changes in cytosolic Ca^2+^ levels ([Ca^2+^]_i_) in different cells in human pancreas slices (e.g., endocrine, vascular, and immune cells; **Figure 2D**). This platform is, thus, very valuable to study intercellular communication in human islets [e.g (28)]. Importantly, we can now simultaneously record changes in vessel diameter and [Ca^2+^]_i_ in adjacent vascular cells in the human islet.

### Human and Mouse Islet Blood Vessels Constrict and Dilate in Response to Different Vasoactive Substances

**Figure 3:**
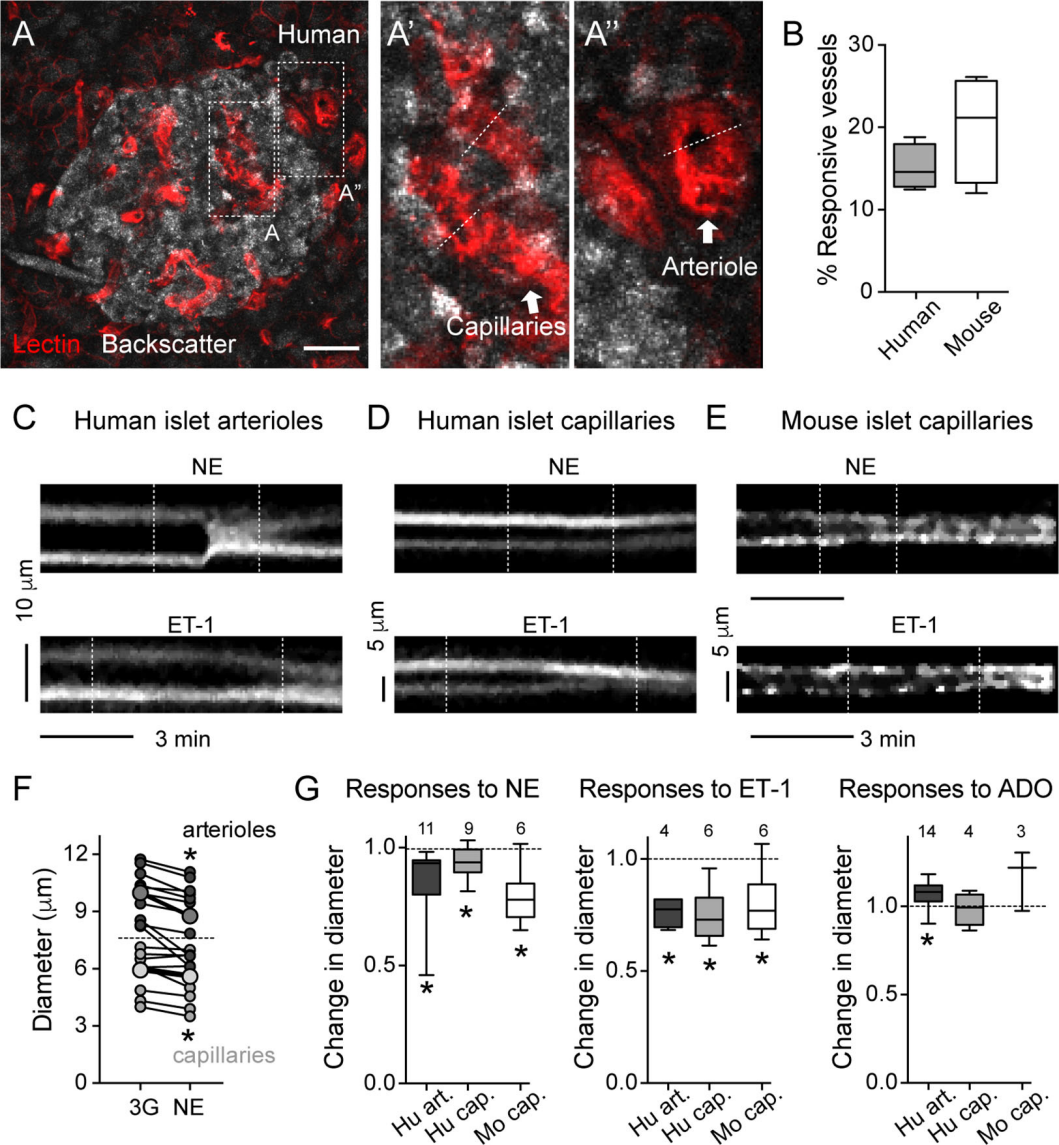
Human and mouse islet blood vessels constrict and dilate in response to different vasoactive substances. **(A)** Maximal projection of confocal images of an islet within a living human pancreas slice. Different types of blood vessels are labeled with a fluorescent lectin (red): capillaries within the islet parenchyma **(A’)** and arterioles at the islet border **(A”)**. Islet endocrine cells are visualized with backscatter. Dashed lines show vessel regions where temporal projections like those shown in **(C–E)** were taken from. Scale bar = 20 μm. **(B)** Quantification of the percentage of the islet microvasculature that shows vasomotion, calculated as the percentage of responsive vessel area divided by the total islet lectin-labeled vessel area [N = 6 mouse islets (from 4 mice); N = 4 human islets (from 4 donors)]. **(C**–**E)** Temporal projections showing changes in diameter induced by norepinephrine (20 μM, NE; upper panels) and endothelin-1 (10 nM, ET-1; lower panels) of human islet arterioles [**C**; region A” showed in **(A)**], human islet capillaries [**D**; region A’ showed in **(A)**] and mouse islet capillaries **(E)**. Vertical dashed lines indicate when stimuli were applied. Stimuli were applied in 3 mM glucose solutions. Both stimuli are vasoconstrictor. Interestingly, in human islets, ET-1-induced capillary constriction starts before arteriole constriction (arteriole and capillary projections are from the same recording). **(F)** Norepinephrine-induced changes in human islet arterioles (dark gray symbols) and capillary diameters (light gray symbols). Shown are diameter values of different vessels right before (3G) and 3 min after norepinephrine (NE). Bigger symbols show the average vessel diameter. *p < 0.05 (paired t-test; N = 9 capillaries, 11 arterioles from 4 different non-diabetic donors). **(G)** Quantification of relative changes in diameter of mouse islet capillaries (white), human islet arterioles (dark gray) and capillaries (light gray box-plot) induced by NE, ET-1 and adenosine (50 μM, ADO) normalized to the initial diameter (after perfusing slices for 3 min with 3 mM glucose solution). Diameter values were taken 3 min after perfusing with norepinephrine (20 μM, NE), 5 min with endothelin-1 (10 nM, ET-1) and 4 min with adenosine (50 μM, ADO). The numbers of vessels analyzed are shown above the box-plots. Data are from 4 different non-diabetic individuals or 3 different mice, at least 1 islet per individual. *p < 0.05 [One sample t-test compared to a theoretical mean of 1 (diameter values were normalized to initial vessel diameter)]. Mouse data in this figure were taken from (11, 22).

**Figure 4:**
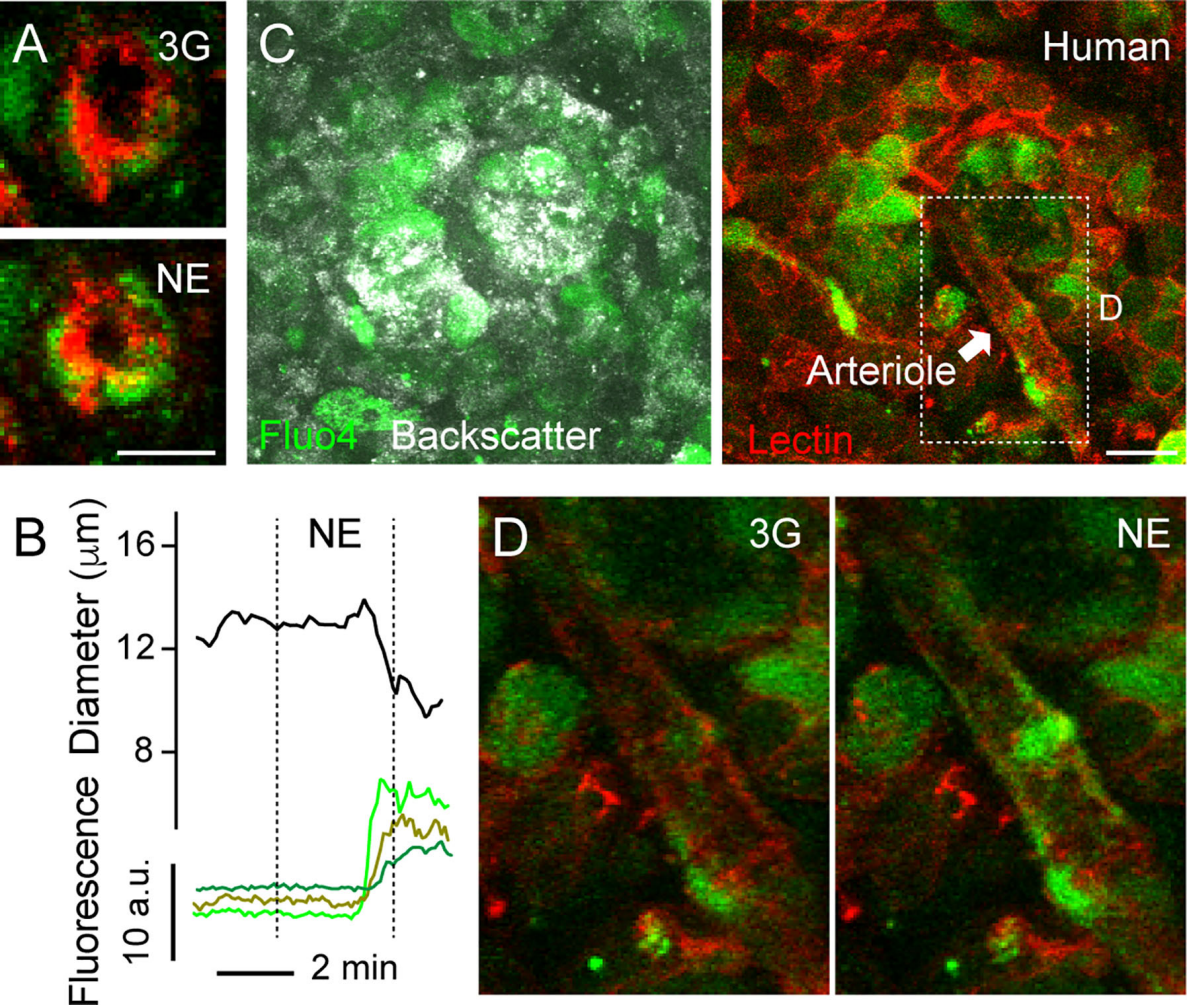
Increases in [Ca^2+^]_i_ in mural cells covering human islet arterioles accompanies vasoconstriction. **(A)** Zoomed confocal images of the islet arteriole shown in (A” in **Figure 3**) before (3G; upper panel) and 3 min after NE application (NE, lower panel). Lectin-labeled vessel is shown in red and cells labeled with calcium indicator Fluo4 are shown in green. Scale bar = 10 μm. **(B)** Traces showing absolute changes in diameter and [Ca^2+^]_i_ in mural cells of the islet feeding arteriole shown in **(A)**, elicited by NE (20 μM). Curve showing changes in diameter over time was smoothened by averaging 4-neighboring values. **(C)** Maximal projection of confocal images of a human islet in a living pancreas slice labeled with Fluo4 (green) and with a lectin (red). The islet feeding arteriole is indicated with an arrow. Endocrine cells in the islet (seen with backscatter) and mural cells on the surface of the feeding arteriole incorporate the calcium indicator. Scale bar = 20 μm. **(D)** Zoomed images of region within dashed rectangle shown in **(C)** showing an increase in [Ca^2+^]_i_ in mural cells covering islet arteriole and vessel constriction triggered by NE.

As previously published and shown in **Figures 1** and **2**, the human islet microvasculature consists of one (or more) feeding arterioles that penetrate the islet and branch off into a network of capillaries that irrigates the whole islet parenchyma (16). Blood leaves the islet through small venules that often drain directly into capillaries in the exocrine tissue [islet-acinar portal system; (41)]. In this study, we did not examine any postcapillary vessel but focused instead on the arteriolar and capillary segments of the islet microvasculature. In many islets in pancreas slices labeled with lectin, we could distinguish a *feeding arteriole*, that is a vessel with a diameter of around 10 μm (8–12 μm) at the islet border (**Figures 1B** and **3A**) or entering the islet parenchyma (**Figures 1C**, **2C**, and **4C**). We considered *islet capillaries* those lectin-labeled vessels surrounded by endocrine cells (cells with backscatter) with a diameter around 5–7 μm [(42); **Figures 3A**and **3A**’].

Several mechanisms have evolved that regulate islet blood perfusion (8). These include signals from neighboring endothelial cells or metabolically active endocrine cells and extrinsic signals, for instance, from the nervous system (16). We have previously observed that mouse islet capillaries are responsive to these islet intrinsic and extrinsic inputs (11). Using living human pancreas slices, we investigated whether these different signals triggered vasomotion in the human islet. Of note, only a subset of islet capillaries (15–20%) exhibited vasomotion in mouse and human islets and was responsive to applied stimuli *ex vivo* (**Figure 3B**). Norepinephrine is endogenously released by sympathetic nerves that innervate blood vessels in the human islet (35). Exogenous norepinephrine (NE, 20 μM) administration significantly constricted the feeding arteriole in human islets (**Figures 3C, F, G**; average reduction of arteriole diameter ~12%) and a subset of human and mouse islet capillaries (5% reduction in human islet capillary diameter; **Figures 3D–G**). Endothelins are vasoconstrictor peptides endogenously produced by endothelial cells (43). Exogenous administration of endothelin 1 (ET-1, 10 nM) led to powerful constriction of human islet feeding arterioles, as well as of human and mouse islet capillaries (**Figures 3C–E, G**). Interestingly, in human islets, capillary constriction induced by ET-1 occurred before arteriole constriction (**Figures 3C, D**); **Supplementary movie S1**). Adenosine is an important mediator of the metabolic blood flow regulation in many tissues. We had previously published that adenosine, endogenously produced from ATP co-released with insulin, mediates the increase in islet capillary diameter and blood flow upon stimulation of mouse islet beta cells with high glucose (11). Regarding human islets, exogenous adenosine administration (ADO, 50 μM) dilated human islet arterioles by 10% and had no significant effect on human islet capillary diameter (**Figure 3G**).

### Vasoactive Substances Change [Ca^2+^]_i_ Levels in Human and Mouse Islet Mural Cells

Our next goal was to better characterize the mechanisms that induce vasomotion in human islets, similarly to what we had done in the mouse. An increase or a decrease in [Ca^2+^]_i_ in mural cells (vascular smooth muscle cells and pericytes) mediate vasoconstriction or vasodilation, respectively (44). Briefly, initiation of a vasoconstrictor myogenic response, for instance, involves an increase in intracellular free calcium levels in mural cells and calmodulin-dependent activation of myosin light chain kinase. This kinase, in turn, phosphorylates myosin light chain and stimulates the formation of cross bridges between myosin and actin filaments, leading to vasoconstriction (45). By incubating living human pancreas slices with a membrane permeant calcium indicator (Fluo4), we noticed that changes in human islet vessel diameter were accompanied by changes in [Ca^2+^]_i_ in neighboring mural cells (**Figure 4**). For instance, NE-mediated arteriole constriction was preceded by an increase in [Ca^2+^]_i_ in cells wrapping this vessel (**Figures 4A, B, D**).

**Figure 5:**
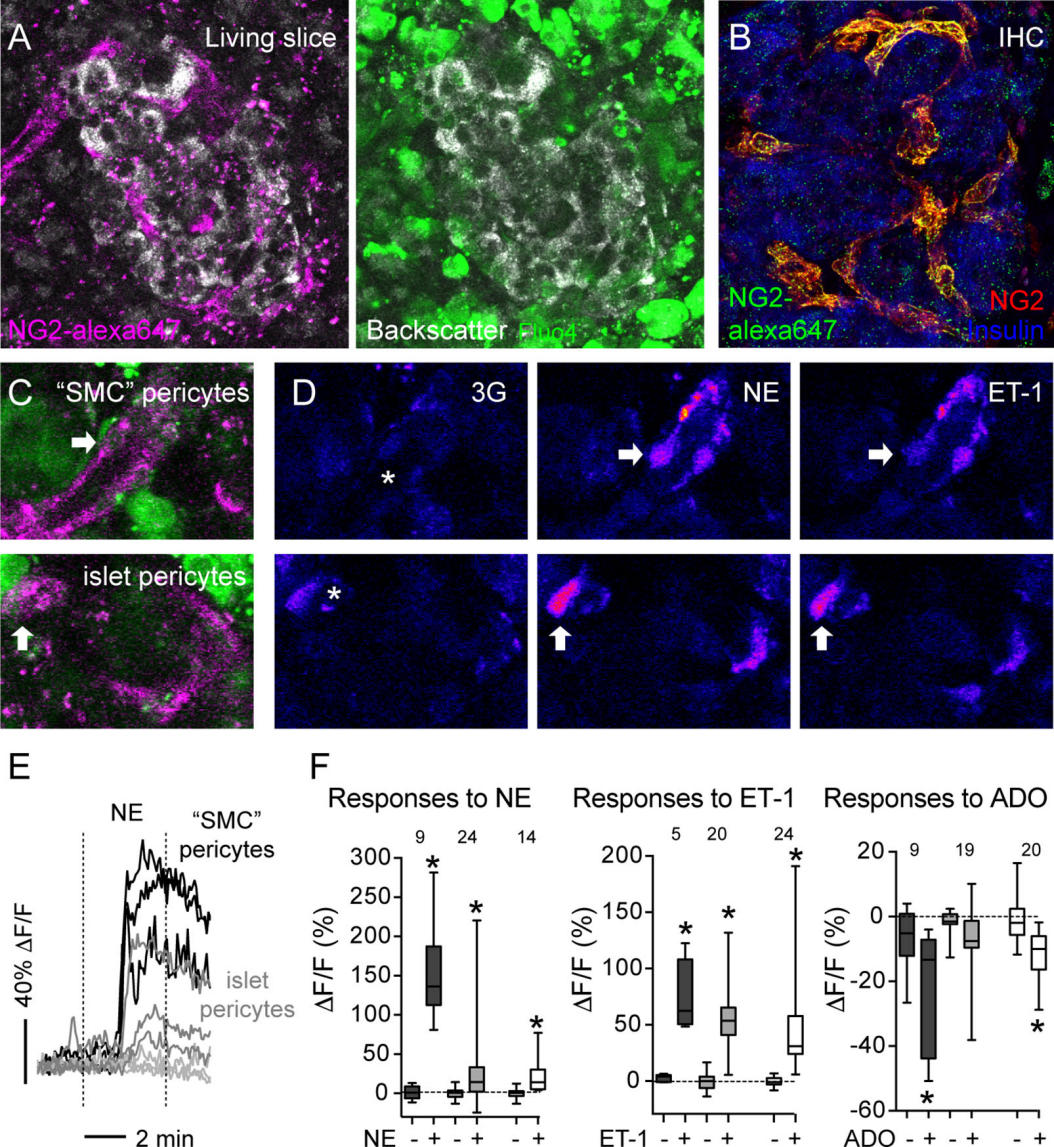
Vasoactive substances change [Ca^2+^]_i_ levels in human and mouse islet mural cells. **(A)** Confocal images of a human islet in a living pancreas slice showing fluorescent NG2 antibody (NG2-alexa647; magenta) labeled pericytes *in situ* and fluo-4 loaded cells (green) in living slices. NG2 is a proteoglycan expressed at the plasma membrane of pericytes. Slices Scale bars = 20 μm. **(B)** Maximal projection of confocal images of a human islet in a pancreatic section immunostained for insulin (blue) and pericytes using two different anti-NG2 antibodies: a non-conjugated one (red; used in **Figure 1**) and another conjugated to Alexa647 (green). NG2-alexa647 recognizes pericytes. **(C)** Confocal images of regions within the human islet shown in **(A)**. Different islet mural cells incorporate the calcium indicator Fluo4 (green) and can be visualized with NG2-alexa647 antibody (magenta), such as “SMC” pericytes (upper panel) and islet pericytes (lower panel). **(D)** Changes in Fluo4 fluorescence of cells shown in **(C)** reflecting changes in [Ca^2+^]_i_ in “SMC” pericytes (upper panels) and islet pericytes (lower panels) under basal conditions (3 mM glucose concentration, 3G, left panels), after 3 min with NE (middle panels) and 5 min with ET-1 (right panels). Pseudocolor (LUT fire) images are shown to better illustrate absolute changes in fluorescence. Arrows indicate different cells responding to the stimuli, and the * shows the vessel lumen. **(E)** Traces showing relative changes in fluorescence induced by norepinephrine (NE) in individual islet mural cells: “SMC” pericytes (black traces), pericytes at the islet border (dark gray traces) and pericytes in the islet parenchyma (light gray traces). **(F)** Quantification of changes in fluorescence induced by NE, ET-1 or adenosine (ADO) for individual mural cells in human and mouse islets: “SMC” pericytes in human islets (dark gray box-plot), human islet pericytes (light gray box-plot) and mouse islet pericytes (white box-plot). Maximum (peak) amplitude values were taken before (-) and at the end of stimulus application (+). [Ca^2+^]_i_ data of mouse islet pericytes was from experiments using mice that expressed the genetically encoded calcium indicator GCaMP3 in pericytes (11). The numbers of cells analyzed are shown above the box-plots. Data are from three different non-diabetic individuals, three different mice, at least one islet per individual. *p < 0.05 (paired t-test, comparisons with corresponding values before stimulus application).

Using transgenic mice that express GCaMP3 in pericytes (GCaMP3 expression is controlled by the NG2 promoter), we had previously determined that pericytes control capillary diameter in mouse islets (11). To determine the nature of vascular cells whose activation accompanied vasoconstriction in human islets, we labeled them *in situ* using an anti-NG2 antibody conjugated to a fluorophore (alexa 647; **Figure 5A**). The pattern of labeling using this fluorescent antibody is very similar to the one achieved using a non-conjugated NG2 antibody (used in **Figure 1**; **Figure 5B**). Human islet pericytes can now be visualized *in situ* in living pancreas slices (**Figures 5A, C**). In particular, as described in **Figure 1**, this antibody labels not only pericytes in the islet parenchyma (islet pericytes) but also cells at the islet border that resemble more smooth muscle cells and that we had named “SMC” pericytes (**Figure 5C**). Islet pericytes and “SMC” pericytes incorporated well the calcium indicator, allowing us to compare their physiological responses to different agonists (**Figure 5C**). We recorded changes in [Ca^2+^]_i_ induced by norepinephrine, endothelin-1 and adenosine in mural cells in human islets and compared with previously published mouse mural cell [Ca^2+^]_i_ data. Norepinephrine (NE) induced a robust and uniform increase in [Ca^2+^]_i_ in “SMC” pericytes that coincide with a reduction of the arteriole diameter (**Figures 5D–F** and **Supplementary movie S2**). Indeed, the response of “SMC” pericytes displayed the synchronicity that is needed for vasomotion (46). NE induced a smaller increase in [Ca^2+^]_i_ in human and mouse islet pericytes. Interestingly, in human islets, pericytes that responded to NE were located at the islet border and pericytes within the islet parenchyma did not respond to the sympathetic agonist (**Figure 5E**). Endothelin-1 produced a very sharp and significant increase in [Ca^2+^]_i_ in mouse and human islet pericytes, as well as in “SMC” pericytes in human islets (**Figures 5D, F**). In human islets, pericytes at the islet border or in the islet parenchyma responded to endothelin-1 (not shown). Adenosine, in turn, was inhibitory and significantly decreased [Ca^2+^]_i_ in mouse islet pericytes, as well as in human islet pericytes and “SMC” pericytes (**Figure 5F**). Our data confirm that pericytes are a very heterogenous population (47) that exhibit various response profiles to different vasoactive substances. The subtype of islet pericyte may be dictated by their location in the islet and differential interactions with other cells within the islet microenvironment, which also differs between mouse and human islets.

## Discussion

In this study, we performed the first detailed functional characterization of the human islet microvasculature, comparing it side-by-side with what we know about the physiology of mouse islet blood vessels. In human islets, a subset of islet pericytes and smooth muscle cell-like (“SMC”) pericytes express the contractile protein αSMA. We measured vasomotion in different types of islet blood vessels in response to exogenous administration of norepinephrine, endothelin-1, and adenosine. We found that islet feeding arterioles and a small subset of capillaries constrict upon norepinephrine and endothelin-1, but only arterioles dilate upon adenosine. We further determined that changes in blood vessel diameter are coupled to changes in cytosolic calcium levels in adjacent pericytes or “SMC” pericytes. Our study points to pericytes as targets of several regulatory mechanisms of islet blood perfusion, similarly to their role in mouse islets.

Our data support that blood flow in the human islet can be regulated locally at the level of the feeding arteriole and of a subset (~15–20%) of islet capillaries, similar to what has been described in rodents (11, 48–50). Here we show that changes in the activity of islet pericytes and “SMC” pericytes mediate vasomotion in human islets and propose that these cells function as local gates. Given recent functional evidence in mice that the islet microcirculation is open and not isolated from that of the surrounding exocrine tissue (51), and the fact that in humans an islet-portal circulatory system is also well developed (41), the existence of such a gating system would allow islet blood flow to be regulated independently of the exocrine tissue under certain conditions, as previously suggested (52).

In this study we perform a functional and anatomical cross comparison of the islet microvasculature between mice and humans. Previous studies had already shown that the architecture and density of islet vascular trees differed significantly between these two species (13, 20). Our analysis has revealed that also the morphology of islet pericytes and blood vessels is more heterogenous in human islets than in mouse islets. Human islets contain a subset of pericytes whose circular cytoplasmic processes give them a smooth muscle cell-like morphology and, therefore, we named them “SMC” pericytes. These type of pericytes localize on the surface of islet feeding arterioles (larger blood vessels that enter the islet parenchyma). Given their location and calcium response profile, “SMC” pericytes resemble precapillary sphincters. The existence of a sphincter-like mechanism had already been reported in monkey islets transplanted into the anterior chamber of the monkey eye (53). Here we show that exogenous norepinephrine induces a stronger activation of islet “SMC” pericytes than of mouse and human islet pericytes and a concomitant and powerful constriction of human islet feeding arterioles. Norepinephrine is a neurotransmitter released at sympathetic nerve terminals. While in mouse islets sympathetic axons equally innervate islet blood vessels and endocrine cells at the periphery, in human islets sympathetic nerves preferentially contact contractile elements around islet blood vessels (35). Thus, sympathetic modulation of islet hormone secretion in humans may occur in part by affecting islet blood flow (54). Disruption of this mechanism may contribute, for instance, to the impaired glucagon secretion characteristic of type 1 diabetics (55), as loss of islet sympathetic nerves has been seen in mouse models of type 1 diabetes (56) and in type 1 diabetic patients (57).

We further show that mouse and human islet blood vessels and their mural cells are very responsive to the vasoconstrictor peptide endothelin-1. Previous study in rats had shown that endothelin-1 induces a pronounced constriction of islet arterioles and decreased islet blood flow, mainly through ET_A_ receptors expressed on smooth muscle cells (58). Interestingly, alterations in endothelin-1 release and action have been consistently shown in diabetic patients and animal models of the disease (59). In future studies, we will explore if similar alterations occur at the level of the islet and contribute to impaired hormone secretion during diabetes pathogenesis.

Blood perfusion in the islet is under tight regulatory mechanisms as it can strongly impact the final hormonal output of the islet. Therefore, several mechanisms have evolved that regulate islet blood flow, combining local paracrine signals (e.g., from neighboring endothelial or endocrine cells) with those from the nervous system or the systemic circulation (8, 16). Although *in vivo* studies are critical to understand the physiological context, *ex vivo* functional studies are essential to elucidate the cellular mechanisms responsible for regulating vascular diameter in human islets (46). Using living pancreas slices from donors without diabetes, we have given the first steps exploring what controls vasomotion in human islets. Similar studies can now be performed with tissue from individuals with diabetes and at different disease stages to assess the functional impact of structural alterations of the islet microvasculature that progressively occur, contributing to our understanding of disease pathogenesis.

## Data Availability Statement

The raw data supporting the conclusions of this article will be made available by the authors, without undue reservation.

## Ethics Statement

The studies involving human participants were reviewed and approved by IRB approval, University of Miami. Written informed consent for participation was not required for this study in accordance with the national legislation and the institutional requirements.

## Author Contributions

LG and JA designed the study, and acquired and analyzed data. Both authors discussed the results and interpreted the data. JA wrote the manuscript. All authors contributed to the article and approved the submitted version.

## Funding

This work was funded by NIH grants K01DK111757 (JA) and by the NIDDK-supported Human Islet Research Network (HIRN, RRID : SCR_014393; https://hirnetwork.org; UC4 DK104162, New Investigator Pilot Award to Joana Almaça).

## Conflict of Interest

The authors declare that the research was conducted in the absence of any commercial or financial relationships that could be construed as a potential conflict of interest.

## References

[1] Bonner-Weir S Orci L New perspectives on the microvasculature of the islets of Langerhans in the rat. Diabetes (1982) 31:883–9. 10.2337/diabetes.31.10.883 6759221

[2] Brissova M Shostak A Shiota M Wiebe PO Poffenberger G Kantz J Pancreatic islet production of vascular endothelial growth factor–a is essential for islet vascularization, revascularization, and function. Diabetes (2006) 55:2974–85. 10.2337/db06-0690 17065333

[3] Lammert E Cleaver O Melton D Induction of pancreatic differentiation by signals from blood vessels. Sci (New York NY) (2001) 294:564–7. 10.1126/science.1064344 11577200

[4] Lammert E Gu G McLaughlin M Brown D Brekken R Murtaugh LC Role of VEGF-A in vascularization of pancreatic islets. Curr Biol CB (2003) 13:1070–4. 10.1016/S0960-9822(03)00378-6 12814555

[5] Sasson A Rachi E Sakhneny L Baer D Lisnyansky M Epshtein A Islet Pericytes Are Required for beta-Cell Maturity. Diabetes (2016) 65:3008–14. 10.2337/db16-0365 27388217

[6] Epshtein A Rachi E Sakhneny L Mizrachi S Baer D Landsman L Neonatal pancreatic pericytes support beta-cell proliferation. Mol Metab (2017) 6:1330–8. 10.1016/j.molmet.2017.07.010 PMC5641631 29031732

[7] Park HS Kim HZ Park JS Lee J Lee SP Kim H beta-Cell-Derived Angiopoietin-1 Regulates Insulin Secretion and Glucose Homeostasis by Stabilizing the Islet Microenvironment. Diabetes (2019) 68:774–86. 10.2337/db18-0864 30728183

[8] Ballian N Brunicardi FC Islet vasculature as a regulator of endocrine pancreas function. World J Surg (2007) 31:705–14. 10.1007/s00268-006-0719-8 17347899

[9] Richards OC Raines SM Attie AD The role of blood vessels, endothelial cells, and vascular pericytes in insulin secretion and peripheral insulin action. Endocrine Rev (2010) 31:343–63. 10.1210/er.2009-0035 PMC3365844 20164242

[10] Almaça J Caicedo A Landsman L Beta cell dysfunction in diabetes: the islet microenvironment as an unusual suspect. Diabetologia (2020) 63:2076–85. 10.1007/s00125-020-05186-5 PMC7655222 32894318

[11] Almaca J Weitz J Rodriguez-Diaz R Pereira E Caicedo A The Pericyte of the Pancreatic Islet Regulates Capillary Diameter and Local Blood Flow. Cell Metab (2018) 27:630–44.e4. 10.1016/j.cmet.2018.02.016 29514070 PMC5876933

[12] Bogdani M Johnson PY Potter-Perigo S Nagy N Day AJ Bollyky PL Hyaluronan and hyaluronan-binding proteins accumulate in both human type 1 diabetic islets and lymphoid tissues and associate with inflammatory cells in insulitis. Diabetes (2014) 63:2727–43. 10.2337/db13-1658 PMC4113060 24677718

[13] Brissova M Shostak A Fligner CL Revetta FL Washington MK Powers AC Human Islets Have Fewer Blood Vessels than Mouse Islets and the Density of Islet Vascular Structures Is Increased in Type 2 Diabetes. J Histochem Cytochem Off J Histochem Soc (2015) 63:637–45. 10.1369/0022155415573324 PMC4530394 26216139

[14] Canzano JS Nasif LH Butterworth EA Fu DA Atkinson MA Campbell-Thompson M Islet Microvasculature Alterations With Loss of Beta-cells in Patients With Type 1 Diabetes. J Histochem Cytochem Off J Histochem Soc (2019) 67:41–52. 10.1369/0022155418778546 PMC6309032 29771178

[15] Gepts W Lecompte PM The pancreatic islets in diabetes. Am J Med (1981) 70:105–15. 10.1016/0002-9343(81)90417-4 7006384

[16] Jansson L Carlsson PO Pancreatic Blood Flow with Special Emphasis on Blood Perfusion of the Islets of Langerhans. Compr Physiol (2019) 9:799–837. 10.1002/cphy.c160050 30892693

[17] St Clair JR Ramirez D Passman S Benninger RKP Contrast-enhanced ultrasound measurement of pancreatic blood flow dynamics predicts type 1 diabetes progression in preclinical models. Nat Commun (2018) 9:1742. 10.1038/s41467-018-03953-y 29717116 PMC5931596

[18] Gepts W The islet of Langerhans: Biochemistry, Physiology and Pathology. Academic Press (1981), Chapter 13. 10.1016/B978-0-12-187820-7.50019-X

[19] Hayden MR Karuparthi PR Habibi J Lastra G Patel K Wasekar C Ultrastructure of islet microcirculation, pericytes and the islet exocrine interface in the HIP rat model of diabetes. Exp Biol Med (Maywood NJ) (2008) 233:1109–23. 10.3181/0709-RM-251 PMC2674965 18641056

[20] Cohrs CM Chen C Jahn SR Stertmann J Chmelova H Weitz J Vessel Network Architecture of Adult Human Islets Promotes Distinct Cell-Cell Interactions In Situ and Is Altered After Transplantation. Endocrinology (2017) 158:1373–85. 10.1210/en.2016-1184 28324008

[21] Virtanen I Banerjee M Palgi J Korsgren O Lukinius A Thornell LE Blood vessels of human islets of Langerhans are surrounded by a double basement membrane. Diabetologia (2008) 51:1181–91. 10.1007/s00125-008-0997-9 18438639

[22] Mateus Gonçalves L Pereira E Werneck de Castro JP Bernal-Mizrachi E Almaça J Islet pericytes convert into profibrotic myofibroblasts in a mouse model of islet vascular fibrosis. Diabetologia (2020) 63:1564–75. 10.1007/s00125-020-05168-7 PMC7354906 32424539

[23] Westermark P Engstrom U Johnson KH Westermark GT Betsholtz C Islet amyloid polypeptide: pinpointing amino acid residues linked to amyloid fibril formation. Proc Natl Acad Sci U S A (1990) 87:5036–40. 10.1073/pnas.87.13.5036 PMC54256 2195544

[24] Westermark P Wilander E The influence of amyloid deposits on the islet volume in maturity onset diabetes mellitus. Diabetologia (1978) 15:417–21. 10.1007/BF01219652 367856

[25] Marciniak A Cohrs CM Tsata V Chouinard JA Selck C Stertmann J Using pancreas tissue slices for in situ studies of islet of Langerhans and acinar cell biology. Nat Protoc (2014) 9:2809–22. 10.1038/nprot.2014.195 25393778

[26] Panzer JK Hiller H Cohrs CM Almaça J Enos SJ Beery M Pancreas tissue slices from organ donors enable in situ analysis of type 1 diabetes pathogenesis. JCI Insight (2020) 5(8):e134525. 10.1172/jci.insight.134525 PMC7205437 32324170

[27] Qadir MMF Álvarez-Cubela S Weitz J Panzer JK Klein D Moreno-Hernández Y Long-term culture of human pancreatic slices as a model to study real-time islet regeneration. Nat Commun (2020) 11:3265. 10.1038/s41467-020-17040-8 32601271 PMC7324563

[28] Weitz JR Jacques-Silva C Fahd Qadir MM Umland O Pereira E Qureshi F Secretory Functions of Macrophages in the Human Pancreatic Islet are Regulated by Endogenous Purinergic Signaling. Diabetes (2020) 69(6):1206–18. 10.2337/db19-0687 PMC7243286 32245801

[29] Houtz J Borden P Ceasrine A Minichiello L Kuruvilla R Neurotrophin Signaling Is Required for Glucose-Induced Insulin Secretion. Dev Cell (2016) 39:329–45. 10.1016/j.devcel.2016.10.003 PMC5123838 27825441

[30] Tang SC Jessup CF Campbell-Thompson M The Role of Accessory Cells in Islet Homeostasis. Curr Diabetes Rep (2018) 18:117. 10.1007/s11892-018-1096-z PMC8011426 30267158

[31] Bergers G Song S The role of pericytes in blood-vessel formation and maintenance. Neuro-oncology (2005) 7:452–64. 10.1215/S1152851705000232 PMC1871727 16212810

[32] Krueger M Bechmann I CNS pericytes: concepts, misconceptions, and a way out. Glia (2010) 58:1–10. 10.1002/glia.20898 19533601

[33] Attwell D Mishra A Hall CN O’Farrell FM Dalkara T What is a pericyte? J Cereb Blood Flow Metab Off J Int Soc Cereb Blood Flow Metab (2016) 36:451–5. 10.1177/0271678X15610340 PMC4759679 26661200

[34] Fowler JL Lee SS Wesner ZC Olehnik SK Kron SJ Hara M Three-Dimensional Analysis of the Human Pancreas. Endocrinology (2018) 159:1393–400. 10.1210/en.2017-03076 PMC5839749 29390052

[35] Rodriguez-Diaz R Abdulreda MH Formoso AL Gans I Ricordi C Berggren PO Innervation patterns of autonomic axons in the human endocrine pancreas. Cell Metab (2011) 14:45–54. 10.1016/j.cmet.2011.05.008 21723503 PMC3135265

[36] Bandopadhyay R Orte C Lawrenson JG Reid AR De Silva S Allt G Contractile proteins in pericytes at the blood-brain and blood-retinal barriers. J Neurocytol (2001) 30:35–44. 10.1023/A:1011965307612 11577244

[37] Parr EL Bowen KM Lafferty KJ Cellular changes in cultured mouse thyroid glands and islets of Langerhans. Transplantation (1980) 30:135–41. 10.1097/00007890-198008000-00012 7010709

[38] Nyqvist D Köhler M Wahlstedt H Berggren PO Donor islet endothelial cells participate in formation of functional vessels within pancreatic islet grafts. Diabetes (2005) 54:2287–93. 10.2337/diabetes.54.8.2287 16046293

[39] Speier S Rupnik M A novel approach to in situ characterization of pancreatic beta-cells. Pflugers Archiv Eur J Physiol (2003) 446:553–8. 10.1007/s00424-003-1097-9 12774232

[40] Ilegems E van Krieken PP Edlund PK Dicker A Alanentalo T Eriksson M Light scattering as an intrinsic indicator for pancreatic islet cell mass and secretion. Sci Rep (2015) 5:10740. 10.1038/srep10740 26030284 PMC5377231

[41] Murakami T Hitomi S Ohtsuka A Taguchi T Fujita T Pancreatic insulo-acinar portal systems in humans, rats, and some other mammals: scanning electron microscopy of vascular casts. Microscopy Res Technique (1997) 37:478–88. 10.1002/(SICI)1097-0029(19970601)37:5/6<478::AID-JEMT10>3.0.CO;2-N 9220425

[42] Henderson JR Moss MC A morphometric study of the endocrine and exocrine capillaries of the pancreas. *Q J Exp Physiol (Cambridge* . England) (1985) 70:347–56. 10.1113/expphysiol.1985.sp002920 3898188

[43] Davenport AP Hyndman KA Dhaun N Southan C Kohan DE Pollock JS Endothelin. Pharmacol Rev (2016) 68:357–418. 10.1124/pr.115.011833 26956245 PMC4815360

[44] Burdyga T Borysova L Calcium signalling in pericytes. J Vasc Res (2014) 51:190–9. 10.1159/000362687 24903335

[45] Kamm KE Stull JT The function of myosin and myosin light chain kinase phosphorylation in smooth muscle. Annu Rev Pharmacol Toxicol (1985) 25:593–620. 10.1146/annurev.pa.25.040185.003113 2988424

[46] Aalkjær C Boedtkjer D Matchkov V Vasomotion - what is currently thought? Acta Physiol (Oxford England) (2011) 202:253–69. 10.1111/j.1748-1716.2011.02320.x 21518271

[47] Shepro D Morel NM Pericyte physiology. FASEB J Off Publ Fed Am Societies Exp Biol (1993) 7:1031–8. 10.1096/fasebj.7.11.8370472 8370472

[48] Brunicardi FC Stagner J Bonner-Weir S Wayland H Kleinman R Livingston E Microcirculation of the islets of Langerhans. Long Beach Veterans Administration Regional Medical Education Center Symposium. Diabetes (1996) 45:385–92. 10.2337/diab.45.4.385 8603757

[49] Liu YM Guth PH Kaneko K Livingston EH Brunicardi FC Dynamic in vivo observation of rat islet microcirculation. Pancreas (1993) 8:15–21. 10.1097/00006676-199301000-00005 8419903

[50] McCuskey RS Chapman TM Microscopy of the living pancreas in situ. Am J Anat (1969) 126:395–407. 10.1002/aja.1001260402 5369107

[51] Dybala MP Kuznetsov A Motobu M Hendren-Santiago BK Philipson LH Chervonsky AV Integrated Pancreatic Blood Flow: Bidirectional Microcirculation Between Endocrine and Exocrine Pancreas. Diabetes (2020) 69:1439–50. 10.2337/db19-1034 PMC7306124 32198213

[52] Almaça J Caicedo A Blood Flow in the Pancreatic Islet: Not so Isolated Anymore. Diabetes (2020) 69:1336–8. 10.2337/dbi20-0016 PMC7306118 32561621

[53] Diez JA Arrojo EDR Zheng X Stelmashenko OV Chua M Rodriguez-Diaz R Pancreatic Islet Blood Flow Dynamics in Primates. Cell Rep (2017) 20:1490–501. 10.1016/j.celrep.2017.07.039 PMC5575201 28793270

[54] Brunicardi FC Sun YS Druck P Goulet RJ Elahi D Andersen DK Splanchnic neural regulation of insulin and glucagon secretion in the isolated perfused human pancreas. Am J Surg (1987) 153:34–40. 10.1016/0002-9610(87)90198-X 3541657

[55] Bolli G de Feo P Compagnucci P Cartechini MG Angeletti G Santeusanio F Abnormal glucose counterregulation in insulin-dependent diabetes mellitus. Interaction of anti-insulin antibodies and impaired glucagon and epinephrine secretion. Diabetes (1983) 32:134–41. 10.2337/diabetes.32.2.134 6337896

[56] Taborsky GJ Jr Mei Q Hackney DJ Figlewicz DP LeBoeuf R Mundinger TO Loss of islet sympathetic nerves and impairment of glucagon secretion in the NOD mouse: relationship to invasive insulitis. Diabetologia (2009) 52:2602–11. 10.1007/s00125-009-1494-5 19798480

[57] Mundinger TO Mei Q Foulis AK Fligner CL Hull RL Taborsky GJ Jr. Human Type 1 Diabetes Is Characterized by an Early, Marked, Sustained, and Islet-Selective Loss of Sympathetic Nerves. Diabetes (2016) 65:2322–30. 10.2337/db16-0284 PMC4955989 27207540

[58] Lai EY Persson AE Bodin B Kallskog O Andersson A Pettersson U Endothelin-1 and pancreatic islet vasculature: studies in vivo and on isolated, vascularly perfused pancreatic islets. Am J Physiol Endocrinol Metab (2007) 292:E1616–23. 10.1152/ajpendo.00640.2006 17284574

[59] Hopfner RL Gopalakrishnan V Endothelin: emerging role in diabetic vascular complications. Diabetologia (1999) 42:1383–94. 10.1007/s001250051308 10651255

