# Supplementary material for: Functional Characterization of the Human Islet Microvasculature Using Living Pancreas Slices
Source: Front Endocrinol (Lausanne). 2021 Jan 15;11:602519. doi: 10.3389/fendo.2020.602519 (PMC7843926; doi:10.3389/fendo.2020.602519)
Supplement: Supplementary file 3 [file Table_1.docx]

**Supplementary Material**

**Table 1. Characteristics of human donors from whom living human pancreas slices were analyzed in this study**

**
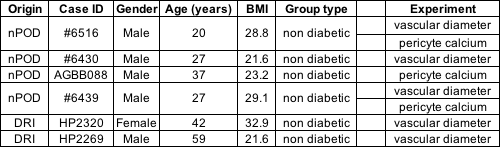
**

Functional data presented in this study (Figures 3, 4 and 5) were obtained with living pancreas slices from the individuals listed above.

**Supplementary movies**

**Movie S1. Responses of human islet blood vessels to endothelin-1.**

Movie composed of a series of maximal projections of confocal images taken every 5 sec of a human islet in a living pancreatic slice labeled with a fluorescent lectin. Reflection (backscattered light) is used to visualize endocrine cells. The lectin labels islet capillaries (vessels surrounded by endocrine cells), an arteriole at the islet border (indicated in the movie with an *) and acinar capillaries (not analyzed in this study). Endothelin-1 (ET-1, 10 nM) constricts a small portion of the islet capillary network (responding vessel indicated with an *) and the islet arteriole (also shown with an *). Endothelin-1 was applied for 5 min. Movie speed 20 frames per second (fps). Related to Figure 3.

**Movie S2. Responses of human islet mural cells to norepinephrine.**

Movie composed of a series of confocal images taken every 5 sec of a human islet in a living pancreas slice. Shown are islet endocrine cells (backscatter) and NG2-alexa647 labeled mural cells (magenta). This antibody labels islet pericytes as well as pericytes transitioning towards a smooth muscle cell phenotype (we named them in the paper “SMC” pericytes) that cover the islet feeding arteriole. Norepinephrine (NE, 20 μM, for 3 min) increases cytosolic Ca^2+^ levels ([Ca^2+^]_i_) in “SMC” pericytes (region indicated with an *) and in islet pericytes located at the islet border (one is shown with an *). Note that increase in [Ca^2+^]_i_ in “SMC” pericytes is uniform and associated with vessel constriction. Movie speed 20 fps. Related to Figure 4.
